# Applying Precision Medicine and Immunotherapy Advances from Oncology to Host-Directed Therapies for Infectious Diseases

**DOI:** 10.3389/fimmu.2017.00688

**Published:** 2017-06-29

**Authors:** Robert N. Mahon, Richard Hafner

**Affiliations:** ^1^Division of AIDS, Columbus Technologies, Inc., Contractor to National Institute of Allergy and Infectious Diseases, National Institutes of Health, Bethesda, MD, United States; ^2^Division of AIDS, National Institute of Allergy and Infectious Diseases, National Institutes of Health, Bethesda, MD, United States

**Keywords:** infectious diseases, immuno-oncology, immunometabolism, precision medicine, tuberculosis, HDT

## Abstract

To meet the challenges of increasing antimicrobial resistance, the infectious disease community needs innovative therapeutics. Precision medicine and immunotherapies are transforming cancer therapeutics by targeting the regulatory signaling pathways that are involved not only in malignancies but also in the metabolic and immunologic function of the tumor microenvironment. Infectious diseases target many of the same regulatory pathways as they modulate host metabolic functions for their own nutritional requirements and to impede host immunity. These similarities and the advances made in precision medicine and immuno-oncology that are relevant for the current development of host-directed therapies (HDTs) to treat infectious diseases are discussed. To harness this potential, improvements in drug screening methods and development of assays that utilize the research tools including high throughput multiplexes already developed by oncology are essential. A multidisciplinary approach that brings together immunologists, infectious disease specialists, and oncologists will be necessary to fully develop the potential of HDTs.

## Introduction

With increasing resistance to current antibiotics and reduced investment in the development of new antimicrobial drug classes, The World Health Organization has warned that we are at risk of entering a “post-antibiotic era” (1). The search for novel therapies to treat infectious diseases is urgent and has led to increased research to develop host-directed therapy (HDT) strategies. Successful pathogens create a hospitable environment within the host and suppress host defenses by altering cellular regulation mechanisms. HDT interventions are intended to enhance microbial killing and lessen detrimental inflammation and tissue damage by targeting host regulatory molecules and pathways modulated by pathogenesis. Preclinical studies have identified drugs with promising HDT benefits (2). The identification of these first-generation HDTs, done primarily in the context of *Mycobacterium tuberculosis* (Mtb) infection, was mainly on an empirical basis. Choosing next-generation HDT candidate agents for evaluation needs to be based on knowledge of the host regulatory signaling pathways that are disrupted by pathogens and identifying specific pathway molecules that can be therapeutically targeted.

Oncology has addressed similar challenges, i.e., reversing disruptions in cell regulatory mechanism that cause malignancies and often lead to suppression of immune processes. In response, oncologic research has developed innovative therapeutic approaches based on discoveries of basic pathogenic mechanisms of malignancies (3). These strategies include “precision medicine” (targeted reversal of cell pathway disruptions caused by mutations as pathogenic drivers) and novel “immuno-oncology” interventions, e.g., immune checkpoint reversal. The same knowledge, tools, and interventions now revolutionizing oncology can be adapted for improved therapy of many infections. These discoveries provide the infectious disease community with a roadmap for possible re-purposing of innovative HDT strategies.

## Precision Medicine—Molecularly Targeted Therapies to Reverse Effects of Microbial Pathogenic Drivers Disrupting Key Regulatory Pathways of Infected Cells

The fundamental principle of precision medicine, to develop interventions specifically targeted at disrupted human cell regulatory control mechanisms, is applicable to infectious diseases. However, the necessary research to utilize these advances for infections has not yet been widely pursued. Quoting Dr. Francis Collins (Director, National Institutes of Health) concerning precision medicine in a commentary, “A New Initiative on Precision Medicine” “… this new understanding of oncogenic mechanisms has begun to influence risk assessment, diagnostic categories, and therapeutic strategies, with increasing use of drugs and antibodies designed to counter the influence of specific molecular drivers. Many targeted therapies have been (and are being) developed, and several have been shown to confer benefits, some of them spectacular.” (4) Pathogens produce their own “molecular drivers” that enhance their survival by subverting host cell regulatory signaling pathways to modify cell metabolism, functions, and internal environment to their advantage. These pathways are critical for maintaining essential functions of cells, including all immune cells. As one example, poly-ADP ribose polymerase activity is targeted by several pathogens including *Helicobacter pylori, Trypanosoma cruzi*, and HIV to enhance their survival (5, 6). Some pathogens also cause epigenetic modifications in infected cells, resulting in longer-term alterations in gene expression. For example, Chlamydia, Legionella, and Mtb possess DNA methyltransferases that decrease expression of immune cell genes critical for effective host defense (7).

Research evaluating the role metabolism plays in controlling immune functions (immunometabolism) has dramatically improved understanding of immune cell differentiation and regulation and lead to identification of novel immunotherapies. In immune cells, AMP-activated protein kinase (AMPK), sirtuins (SIRT), hypoxia-inducible factor-1α (HIF-1α), and mechanistic target of rapamycin (mTOR) are examples of signaling molecules that are essential “metabolic checkpoints” conserved in some form among all eukaryotic cell lineages. They regulate switches between highly active (aerobic glycolysis) and quiescent (oxidative phosphorylation, particularly of fatty acids) metabolic states (8). Other critical immune cell metabolic pathways include those involving the tricarboxylic cycle, glutamine, fatty acid synthesis, amino acids, and pentose phosphate (9). Metabolites, including succinate, produced by these metabolic pathways regulate immunometabolism in tandem with signaling molecules to affect immune activity (10). These metabolic switches occur during differentiation and maturation of many immune cell lineages and facilitate transitions between various functional states, for example, between inflammatory M1 macrophages and anti-inflammatory M2 macrophages, effector and memory CD8^+^ T lymphocytes, and Th1/Th17 versus Th2 CD4^+^ T cells.

**Figure 1:**
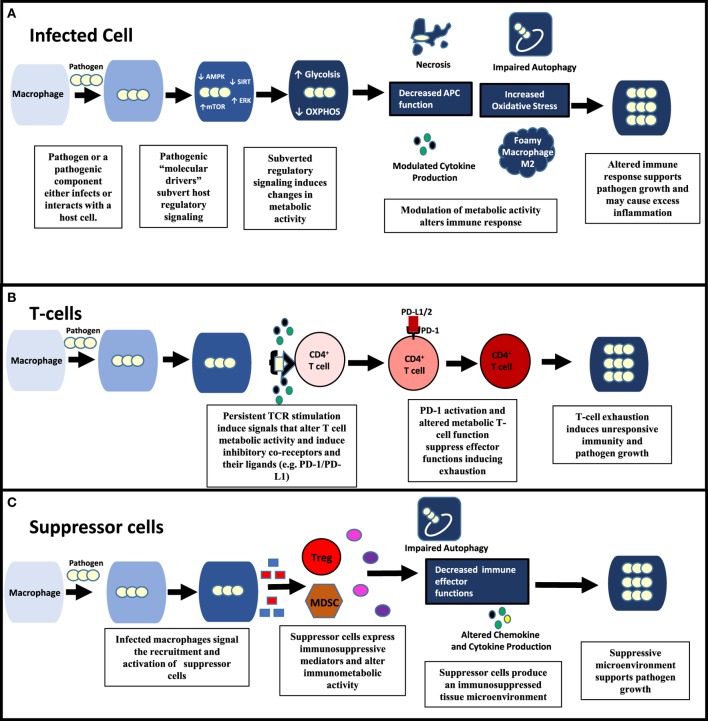
Pathogenic modulation of immunometabolic activity alters all aspects of immune function and pathogen survival. **(A)** Within an infected cell, pathogens possess “molecular drivers” (e.g., lipoproteins and glycolipids) that can modulate signaling of key regulatory pathways (e.g., ERK, AMP-activated protein kinase (AMPK), mechanistic target of rapamycin (mTOR)) involved in energy metabolism. By affecting the metabolic activity of the infected cell, pathogens alter the immune response contributing to their survival. **(B,C)** Pathogens can also modulate uninfected immune cells in the microenvironment. **(B)** By inducing persistent CD4^+^ T cell activation, pathogens cause increased PD-1 expression on CD4 T cells as well as its ligands (e.g., PD-L1) producing an exhausted T cell response. Changes in metabolic activity that occur during both persistent T cell activation and PD-1 signaling significantly contribute to T-cell exhaustion. **(C)** Suppressor cells [e.g., Tregs, myeloid-derived suppressor cells (MDSCs)] are recruited and activated by infected macrophages through the release molecules that include death-associated molecular patterns and pathogen associated molecular patterns (PAMPs) (e.g., HMGB-1, lipoproteins). Suppressor cells produce an immunosuppressive environment through the production of mediators (e.g., ROS, indoleamine-pyrrole 2,3-dioxygenase, and arginase) that induce oxidative stress, apoptosis, and alter immunometabolic activity.

Intracellular pathogens target these metabolic switches to suppress immune function (Figure 1A). HIF-1α, as a master regulator of hypoxia, plays an important role in regulating inflammation during chronic infection. Pathogens such as *Francisella tularensis* and *Pseudomonas aeruginosa* downregulate HIF-1α to inhibit glycolysis and with it inflammatory cytokine production (11, 12). *Streptococcus pyogenes* and Mtb both produce glycohydrolases that deplete nicotinamide adenine dinucleotide (NAD^+^), a key metabolite in macrophage function (13, 14). Additionally, these metabolic switches are targeted by pathogens to re-program host cellular metabolism to meet their nutritional requirements (“pathometabolism”) (15). *Leishmania infantum* utilizes host cell SIRT-1 and AMPK energy sensors to switch cell metabolism from glycolysis to oxidative phosphorylation, a requirement for *L. infantum* survival (16). Other intracellular parasitic infections, including malaria and *Toxoplasma gondii* induce HIF-1α to regulate glycolytic activity and suppress macrophage proliferation (17). Virulent Mtb causes macrophages to differentiate into foam cells by blocking the glycolytic pathway (18).

Drugs targeting these checkpoint molecules, originally designed for treating cancer or metabolic syndromes, are now being tested as HDTs for pathogens. Metformin, largely through an AMPK-dependent pathway, lessens tuberculosis lung pathology by reducing chronic inflammation and tissue pathology and improves bacterial clearance in an animal model (19). Statins have both antimicrobial effects by modulating the ability of pathogens to utilize host cholesterol and immunopotentiating effects, for example, against *Leishmania* (20, 21). In addition to post-infection therapeutics, targeting these molecules and metabolites may also improve vaccine effectiveness through epigenetic changes and trained immunity (22–24). A thorough, though not complete list of candidate HDT agents for infections (all orally administered, non-cytotoxic, and almost all FDA-approved or in clinical trials) has recently been published (25).

Tissue destruction caused by excess inflammation and septic shock are detrimental components of immune responses to severe infections. By targeting signaling molecules such as Akt and mitogen-activated protein kinases to cause immunometabolism and epigenetic modifications, several pathogens including *H. pylori* and *Salmonella* can increase inflammation and necrosis while inhibiting autophagic and apoptotic pathways (26, 27). Agents to enhance autophagy and apoptosis while decreasing necrosis could improve microbial killing and decrease destructive inflammation. One key aspect of HDT target discovery research will be distinguishing between detrimental immune cell regulation changes caused by pathogen molecular drivers and adaptive changes that assist host defense. In some cases, targeted interventions may be used to enhance immune cell regulatory alterations in response to pathogens that result in improved defense, but not to an optimal level.

## Immunotherapy—Therapeutic Strategies to Enhance Antimicrobial Immune Functions of Non-Infected Cells

Current cancer immunotherapy interventions include targeting co-inhibitory T-cell checkpoint receptors, for example, by ipilimumab (CTLA-4) and pembrolizumab (PD-1), to reverse immune exhaustion and improve tumor responses. Various antagonists of suppressive co-receptors and agonists of stimulatory co-receptors, including OX-40 and 4-1BB, are in use or being investigated for treatment of several malignancies and to enhance cancer vaccine responses (28). The regulatory mechanisms of the metabolic and co-receptor immune checkpoints are often interrelated. PD-1 activation alters T-cell metabolic reprogramming by inhibiting glycolysis and promoting lipolysis and fatty acid oxidation (29). The result is increased T-cell longevity at the cost of reduced T-effector cell function. PD-1 antagonists reverse these metabolic changes. While the currently approved co-receptor checkpoint antagonist agents are antibodies, oral drugs that target other co-inhibitory receptors are either approved or in clinical development. For example, adenosine receptor antagonists, including istradefylline now in phase three clinical trials for Parkinson Disease, are being studied as an adjunctive cancer treatment (30).

Pathogens causing chronic infections may also activate inhibitory co-receptor checkpoints resulting in T-cell exhaustion and depressed adaptive immunity (Figure 1B). Early studies with LCMV indicated that by inhibiting PD-1, T cell exhaustion could be reversed. Further work with HIV and other chronic viral infections have shown that combination therapy against multiple co-inhibitory receptors can synergistically reverse T cell exhaustion (31). PD-1 research in the context of Mtb and other intracellular pathogens, though, presents the complexity of the roles of these co-receptors (32, 33). In studies with PD-1-deficient mice, inflammation and pulmonary tuberculosis growth were increased, while survival was decreased. However, *ex vivo* inhibition of PD-1 improved responses of T cells obtained from humans with pulmonary tuberculosis. Adding to the complexity is a recent study indicating that unlike the L1 ligand, the L2 ligand of PD-1 plays an essential role in establishing CD4^+^ T-cell mediated immunity in a malaria model (34). Defining the roles of immune checkpoints during different stages of infections is a major research gap. The roles of co-stimulatory immune cell receptors are also largely unexplored, but could be promising. Stimulation of the OX40 co-stimulatory receptor improved protection from BCG vaccine when administered simultaneously as a fusion protein in the murine model (35).

Also in common with malignant cells, infections induce immune suppressor cells, for example, myeloid-derived suppressor cells (MDSCs) and Tregs. These cells have several immunomodulatory effects, including downregulation of cytokine and chemokine expression, cell trafficking, antigen processing and presentation, and autophagy (Figure 1C). Suppressor cells utilize a variety of mechanisms, such as increased inhibitory reactive oxygen species production, disrupting metabolic activity, and inducing the expression of indoleamine-pyrrole 2,3-dioxygenase (IDO) (36, 37). Pathogens can activate suppressor cells by TLR signaling and death-associated molecular pattern release to improve their survival. Pathogens may also induce suppressor subsets of other immune cell types, including macrophages, neutrophils, and natural killer cells. Therapeutic approaches now utilized in oncology to target these cells include all-trans retinoic acid and inhibitors of mevalonate metabolism (bisphosphonates), IDO, and phosphodiesterase-5 (38).

## The Path Forward

Hotchkiss and Moldawer, in their article, “Parallels between Cancer and Infectious Disease,” point out the similarities in changes in regulation of immune responses to malignancies and infectious pathogens that involve modulation of common signaling pathways. They also noted that immunomodulatory therapies for infections may lead to an over-reactive immune response and excess tissue damage (39). Both preclinical and clinical HDT studies must carefully address and monitor for this risk. Successful adaption of HDT for infections will require careful research to determine which patient subpopulations, types of agent, timing of initiation, and dosing regimens/duration will result in the most benefit and least harm. Development of assays to identify specific immune defects caused by specific infections, including nucleic acid- or proteomic-based detection and quantification of specific mediators of immunosuppression, would help to guide these choices and to monitor immunologic responses to HDT.

To develop such assays, the fundamental regulatory molecular disruptions causing the immunologic defects must be precisely identified. Oncologic research has utilized cutting edge tools for high throughput multiplex (genomic, epigenetic, proteomic, transcriptomic, and metabolomic) analyses of single cells from heterogeneous populations. Studies utilizing these methodologies, including CHiP analyses and DNA methylation mapping of epigenetic factors, will be essential for the identification of targets for further development as HDTs against pathogens.

Support for research to identify these specific immune cell core regulatory pathway disruptions caused by pathogens must be greatly increased to efficiently develop new HDTs for treatment and prevention. Also, drug screening methods are needed to identify which of the dozens of molecularly targeted HDT drug classes now in clinical use or evaluation for other diseases may be effective for infectious diseases. However, current screening methods using *in vitro* cell line infection models have yielded poor results. Improved *in vitro* screening models, for example, human multiple cell type-based 3-D granuloma models for TB, will need to be developed. Re-purposing of HDT drugs now in the clinic for therapy of many non-infectious diseases is a practical approach to address the lack of financial incentive to develop novel antimicrobial agents due to the high cost and lengthy development time needed for marketing approval of new drug classes versus limited financial return.

As pointed out by Dr. Collins, one goal of the precision medicine initiative is to “… instigate the next generation of scientists to develop novel approaches to be applied to a wide range of diseases with multidisciplinary approaches” (4) In order to develop innovative new HDTs for infectious diseases, such multidisciplinary teams of researchers will be needed with expertise in microbiology/clinical infectious diseases, classical immunology, and the still emerging field of molecular biology of cell regulation. The first step in this process is to facilitate collaboration among researchers studying key regulatory signaling pathways and targeted interventions for non-communicable diseases and those in the infectious diseases community. These approaches would create a transformative new paradigm for the treatment and prevention of a wide variety of infectious diseases and have a high global health impact, particularly in the face of progressive development of antimicrobial resistance. However, without meaningful financial support, the pace of development of such crucial research collaborations and the application of these innovative life-saving advances to infectious diseases will remain unacceptably slow.

## Author Contributions

RM and RH contributed equally to the development and writing of the paper, reviewing relevant literature, and preparation of figure in the paper.

## Disclaimer

This manuscript was written by one of the authors as a National Institute of Allergy and Infectious Diseases (NIAID) employee, but the views expressed in this manuscript do not necessarily represent those of NIAID.

## Conflict of Interest Statement

The authors declare that the research was conducted in the absence of any commercial or financial relationships that could be construed as a potential conflict of interest.

## References

[1] Antimicrobial Resistance: Global Report on Surveillance. Geneva, Switzerland: World Health Organization (2014). Available from: http://apps.who.int/iris/bitstream/10665/112642/1/9789241564748_eng.pdf?ua=1

[2] Wallis RS Hafner R. Advancing host-directed therapy for tuberculosis. Nat Rev Immunol (2015) 15:255–63. 10.1038/nri3813 25765201

[3] Palucka AK Coussens LM. The basis of oncoimmunology. Cell (2016) 164:1233–47. 10.1016/j.cell.2016.01.049 26967289 PMC4788788

[4] Collins FS Varmus H. A new initiative on precision medicine. N Engl J Med (2015) 372:793–5. 10.1056/NEJMp1500523 25635347 PMC5101938

[5] Nossa CW Blanke SR. *Helicobacter pylori* activation of PARP-1: usurping a versatile regulator of host cellular health. Gut Microbes (2010) 1:373–8. 10.4161/gmic.1.6.13572 21468218 PMC3056101

[6] Ba X Gupta S Davidson M Garg NJ *Trypanosoma cruzi* induces the reactive oxygen species-PARP-1-RelaA pathway for up-regulation of cytokine expression in cardiomyocytes. J Biol Chem (2010) 285:11596–606. 10.1074/jbc.M109.076984 20145242 PMC2857037

[7] Pereira JM Harmon MA Cossart P A lasting impression: epigenetic memory of bacterial infections. Cell Host Microbe (2016) 19:579–82. 10.1016/j.chom.2016.04.012 27173925

[8] Pearce EL Pearce EJ. Metabolic pathways in immune cell activation and quiescence. Immunity (2013) 38:633–43. 10.1016/j.immuni.2013.04.005 23601682 PMC3654249

[9] O’Neil LA Kishton RJ Rathmell J. A guide to immunometabolism for immunologists. Nat Rev Immunol (2016) 16:553–65. 10.1038/nri.2016.70 27396447 PMC5001910

[10] Tannahi GM Curtis AM Adamik J Palsson-McDermott EM McGettrick AF Goel G Succinate is an inflammatory signal that induces IL-1β through HIF-1α. Nature (2013) 496:238–42. 10.1038/nature11986 23535595 PMC4031686

[11] Wyatt EV Diaz K Griffin AJ Rasmussen JA Crane DD Jones BD Metabolic reprogramming of host cells by virulent *Francisella tularensis* for optimal replication and modulation of inflammation. J Immunol (2016) 196:4227–36. 10.4049/jimmunol.1502456 27029588 PMC4868765

[12] Legendre C Reen FJ Mooij MJ McGlacken GP Adams C O’Gara F *Psuedomonas aeruginosa* alkyl quinolones repress hypoxia-inducibe factor 1 (HIF-1) signaling through HIF-1α degradation. Infect Immun (2012) 80:3985–92. 10.1128/IAI.00554-12 22949552 PMC3486049

[13] Chandrasekaran S Cpaaron MG The *Streptococcus pyogenes* NAD^+^glycohydrolase modulates epithelial cell PARylation and HMGB1 release. Cell Microbiol (2015) 17:1376–90. 10.1111/cmi.12442 25818652 PMC4624300

[14] Sun J Siroy A Lokareddy RK Speer A Doornbos KS Cingolani G The tuberculosis necrotizing toxin kills macrophages by hydrolyzing NAD. Nat Struct Mol Biol (2015) 22:672–8. 10.1038/nsmb.3064 26237511 PMC4560639

[15] Eisenreich W Heesemann J Rudel T Goebel W Metabolic adaptations of intracellular bacterial pathogens and their mammalian host cells during infection (“Pathometabolism”). Microbiol Spectr (2015) 3(3):1–24. 10.1128/microbiolspec.MBP-0002-2014 26185075

[16] Moreira D Rodrigues V Abengozar M Rivas L Rial E Laforge M *Leishmania infantum* modulates host macrophage mitochondrial metabolism by hijacking the SIRT1-AMPK axis. PLoS Pathog (2015) 11:e1004684. 10.1371/journal.ppat.1004684 25738568 PMC4349736

[17] Charpentier T Hammami A Stäger S Hypoxia inducible factor 1α: a critical factor the immune response to pathogens and *Leishmania*. Cell Immunol (2016) 309:42–9. 10.1016/j.cellimm.2016.06.002 27372381

[18] Singh V Kaur C Chaudhary VK Rao KV Chatterjee S. *M. tuberculosis* secretory protein ESAT-6 induces metabolic flux perturbations to drive foamy macrophage differentiation. Sci Rep (2015) 5:12906. 10.1038/srep12906 26250836 PMC5388048

[19] Singhal A Jie L Kumar P Hong GS Leow MK Paleja B Metformin as adjunct antituberculosis therapy. Sci Transl Med (2014) 6(263):263ra159. 10.1126/scitranslmed.3009885 25411472

[20] Hennessy E Adams C Reen FJ O’Gara F Is there potential for repurposing statins as novel antimicrobials. Antimicrob Agents Chemother (2016) 60:5111–21. 10.1128/AAC.00192-16 27324773 PMC4997871

[21] Parihar SP Hartley MA Hurdayal R Guler R Brombacher F Topical simivastatin as host-directed therapy against severity of cutaneous leishmaniasis in mice. Sci Rep (2016) 6:33458 10.1038/srep33458 27632901 PMC5025842

[22] Jayashankar L Hafner R Adjunct strategies for tuberculosis vaccines: modulating key immune cell regulatory mechanisms to potentiate vaccination. Front Immunol (2016) 7:577 10.3389/fimmu.2016.00577 28018344 PMC5159487

[23] Arts RJ Novakovic B Ter Horst R Carvalho A Bekkering S Lachmandas E Glutaminolysis and fumarate accumulation integrate immunometabolic and epigenetic programs in trained immunity. Cell Metab (2016) 24:807–19. 10.1016/j.cmet.2016.10.008 27866838 PMC5742541

[24] Arts RJ Carvalho A La Rocca C Palma C Rodrigues F Silvestre R Immunometabolic pathways in BCG-induced trained immunity. Cell Rep (2016) 17:2562–71. 10.1016/j.celrep.2016.11.011 27926861 PMC5177620

[25] Mahon RN Hafner R. Immune cell regulatory pathways unexplored as host-directed therapeutic targets for *Mycobacterium tuberculosis*: an opportunity to apply precision medicine innovations to infectious diseases. Clin Infect Dis (2015) 61(Suppl 3):S200–16. 10.1093/cid/civ621 26409283 PMC4583576

[26] Robinson KS Aw R. The commonalities in bacterial effector inhibition of apoptosis. Trends Microbiol (2016) 24:665–80. 10.1016/j.tim.2016.04.002 27117049

[27] Conrad M Angeli JP Vandenabeele P Stockwell BR. Regulated necrosis: disease relevance and therapeutic opportunities. Nat Rev Drug Discov (2016) 15:348–66. 10.1038/nrd.2015.6 26775689 PMC6531857

[28] Melero I Berman DM Aznar MA Korman AJ Pérez Gracia JL Haanen J. Evolving synergistic combinations of targeted immunotherapies to combat cancer. Nat Rev Cancer (2015) 15:457–72. 10.1038/nrc3973 26205340

[29] Bengsch B Johsnon AL Kurachi M Odorizzi PM Pauken KE Attanasio J Bioenergetic insufficiencies due to metabolic alterations regulated by the inhibitory receptor PD-1 are an early driver of CD8+ T cell exhaustion. Immunity (2016) 45:358–73. 10.1016/j.immuni.2016.07.008 27496729 PMC4988919

[30] Leone RD Lo YC Powell JD. A2aR antagonists: next generation checkpoint blockade for cancer immunotherapy. Comput Struct Biotechnol J (2015) 13:265–72. 10.1016/j.csbj.2015.03.008 25941561 PMC4415113

[31] Attanasio J Wherry EJ Costimulatory and coinhibitory receptor pathways in infectious disease. Immunity (2016) 44:1052–68. 10.1016/j.immuni.2016.04.022 27192569 PMC4873956

[32] Barber DL Mayer-Barber KD Feng CG Sharpe AH Sher A. CD4 T cells promote rather than control tuberculosis in the absence of PD-1-mediated inhibition. J Immunol (2011) 186:1598–607. 10.4049/jimmunol.1003304 21172867 PMC4059388

[33] Singh A Mohan A Dey AB Mitra DK Inhibiting the programmed death 1 pathway rescues *Mycobacterium tuberculosis*-specific interferon γ-producing T cells from apoptosis in patients with pulmonary tuberculosis. J Infect Dis (2013) 208:603–15. 10.1093/infdis/jit206 23661793

[34] Karunarathne DS Horne-Debets JM Huang JX Faleiro R Leow CY Amante F Programmed death-1 ligand 2-mediated regulation of the PD-L1 to PD-1 axis is essential for establishing CD4^+^ T cell immunity. Immunity (2016) 45:333–45. 10.1016/j.immuni.2016.07.017 27533014

[35] Snelgrove RJ Cornere MM Edwards L Dagg B Keeble J Rodgers A OX40 ligands fusion protein delivered simultaneously with the BCG vaccine provides superior protection against murine *Mycobacterium tuberculosis* infection. J Infect Dis (2012) 205:975–83. 10.1093/infdis/jir868 22315280 PMC3282567

[36] Kumar V Patel S Tcyganov E Gabrilovich DI. The nature of myeloid-derived suppressor cells in the tumor microenvironment. Trends Immunol (2016) 37:208–20. 10.1016/j.it.2016.01.004 26858199 PMC4775398

[37] Josefowicz SZ Lu LF Rudensky AY Regulatory T cells: mechanisms of differentiation and function. Annu Rev Immunol (2012) 30:531–64. 10.1146/annurev.immunol.25.022106.141623 22224781 PMC6066374

[38] De Veirman K Van Valckenborgh E Lahmar Q Geeraerts X De Bruyne E Menu E Myeloid-derived suppressor cells as therapeutic target in hematological malignancies. Front Oncol (2014) 4:349. 10.3389/fonc.2014.00349 25538893 PMC4258607

[39] Hotchkiss RS Moldawer LL Parallels between cancer and infectious disease. N Engl J Med (2014) 371:380–3. 10.1056/NEJMcibr1404664 25054723

